# Erratum

**DOI:** 10.4269/ajtmh.15-0630err

**Published:** 2018-07

In *Am J Trop Med Hyg* 96 (1) by Del Castillo et al (https://doi.org/10.4269/ajtmh.15-0630) there is an error in the dosing. On page 167, in the 4^th^ paragraph under the heading “Case Report” it reads “In consultation with the CDC, the infant was administered loading doses of quinidine and clindamycin, and was subsequently placed on a quinidine drip (10 mg/kg/minute) and a regimen of intravenous (IV) clindamycin (5 mg/kg/dose) every 8 hours.” This should read “... was subsequently placed on a quinidine drip (10 mcg/kg/minute) ...” The journal regrets the error.

